# Fresh and Hardened Properties of Portland Cement-Slag Concrete Activated Using the By-Product of the Liquid Crystal Display Manufacturing Process

**DOI:** 10.3390/ma13194354

**Published:** 2020-09-30

**Authors:** Sung Choi, Sukhoon Pyo

**Affiliations:** 1 Department of Civil Engineering, Kyungdong University, 27 Gyeongdongdaehak-ro, Yangju-Si 11458, Korea; csomy1113@kduniv.ac.kr; 2 Department of Urban and Environmental Engineering, Ulsan National Institute of Science and Technology (UNIST), 50 UNIST-gil, Ulju-gun, Ulsan 44919, Korea

**Keywords:** alternative alkali-activated material, ground granulated blast-furnace slag, strength development, setting time, workability

## Abstract

This experimental research investigated the applicability of the liquid crystal display (LCD) by-product of the refining process as a sustainable and alternative alkali activator for ground granulated blast-furnace slag (GGBFS) blended cement concrete. Three levels of binder replacement using the industrial by-product, and four water/binder ratios were considered in order to evaluate the effects of the replacement in fresh and hardened properties of the blended concrete. XRD and TG analyses confirmed that the by-product that contains abundant alkali compounds promotes the reactivity of GGBFS. The test results indicated that the incorporation of the by-product results in delayed setting and degraded workability due to the highly porous nature of the by-product, yet shows rapid early-age strength development of the blended concrete as conventional alkaline activators for GGBFS. These characteristics shed light on a simple yet effective and practical means of reusing the industrial by-product as an alternative alkaline activator.

## 1. Introduction

As one of the most sustainable approaches to reducing carbon dioxide emissions from the production of cement-based construction materials, various types of industrial by-products are currently used worldwide as Portland cement replacements, called supplementary cementitious materials (SCMs), e.g., ground granulated blast-furnace slag (GGBFS), fly ash, and silica fume. It is well known that the replacement level of cement with SCMs depends on the reactivity, local availability, and legislation [1]. For the strength evolution of SCMs with cement, the pozzolanic reaction of SCMs is an essential chemical process that uses high alkalinity compounds, such as Ca(OH)_2_, from cement hydration products [2]. Although blending cement with SCMs has many advantages—such as reducing carbon dioxide emissions by saving cement, and improving long-term strength, durability, and chemical stability [3,4,5,6,7]—the setting time and early-age strength development can be drastically delayed if the amount SCMs in the blend is excessive, without adequate alkali activators [8,9]. 

Various alkali activators have been suggested for adequately promoting strength of blended concrete using SCMs to either partially or fully replace ordinary Portland cement (OPC). For example, it is well known that sodium hydroxide (NaOH), sodium silicate (Na_2_O·*r*SiO_2_), sodium carbonate (Na_2_CO_3_), and sodium sulphate (Na_2_SO_4_) are effective and common ones used to activate GGBFS [10,11]. These alkali activators are effective in promoting the initial reactivity of GGBFS and lead to rapid strength development [3]. However, the radical chemical reaction can also dramatically accelerate the reaction of GGBFS, which can significantly affect the workability of concrete—such as its setting time and flowability—depending on the alkali content and the slag/activator ratio [10,12]. In addition, the manufacturing process of alkali activators is usually both energy intensive and costly [13], which could limit wider application of alkali activated slag concrete. Alternative studies have been attempted to evaluate sustainable alkali activators for SCMs using industrial by-products [14]. For example, Maraghechi et al. [1] tested recycled glass powder as an alkali activator for binary mixtures with OPC, slag, and fly ash. The findings suggested that elevated temperature curing (60 ℃) is preferable to effectively consuming glass powder for alkali activated slag mortar. However, limited work has been conducted on the use of industrial by-products as alkali activators for slag cement and concrete.

The current mainstream liquid crystal display (LCD) design primarily consists of color filter (CF) substrate glass on which RGB pixels are deposited and of thin-film-transistor (TFT) substrate glass that is painted with thin film circuitry that delivers signals to liquid crystal. In order to maintain a high quality and a high resolution, TFT uses an alkali-free glass that is obtained through a high refining process that involves the generation of by-product. Because the by-product of this refining process contains a large amount of alkali ingredients—such as SO_3_, Na_2_O, and K_2_O—it is expected to be used as an alternative alkali activator for GGBFS blended cement concrete. Although the LCD by-product that results from the refining process does not require an additional treatment because it is in the form of powder, it is necessary to investigate its fundamental properties in order to verify its applicability as an alternative alkali activator for GGBFS blended cement concrete. However, to the best of the authors’ knowledge, alkaline activation of LCD by-product has not been investigated as a means of developing sustainable construction material that can be utilized for the production of high strength concrete. In order to adapt the alkali industrial by-product to slag concrete, it is necessary to find suitable combinations of base binders by investigating the reactivity of the by-product with existing binding materials. Therefore, the effects of the alkali by-product on the fundamental properties of concrete—such as workability, setting time, and strength enhancement under various mixing conditions—need to be identified. This research gap motivated the study presented in this paper. It should be pointed out, however, that multiple attempts have been conducted to use thin-film transistor liquid-crystal display (TFT-LCD) waste glass to partially replace OPC [15,16,17,18]. For example, Lin et al. [15] tested up to 40% of TFT-LCD waste glass to replace OPC, and concluded that as the amount of TFT-LCD waste glass increases the strength of the paste distinctly decreases. Jang et al. [16] used additional activator to promote reactivity of TFT-LCD waste glass with OPC, and concluded that the pozzolanic reaction between the waste glass and the activator leads to enhance compressive strength of high strength concrete products. Kim et al. [18] found that smaller particle size of ground TFT-LCD waste glass would lead to the decreased porosity of TFT-LCD waste glass concrete, which is expected to enhanced durability and permeability.

In this study, the applicability of the LCD by-product of the refining process as an alkali activator was evaluated by characterizing the reactivity of GGBFS with OPC under a normal curing condition with the aim of developing practical applications. The binary paste was prepared by mixing GGBFS and OPC with the LCD by-product-based activator (LCDBA) and the variation of the hydration products in the paste, according to the curing age, was characterized through X-ray diffraction (XRD) as well as through thermogravimetry and differential thermal analysis (TG-DTA). In addition, fresh and hardened properties of OPC-slag concrete, activated by the alkali by-product, were assessed by examining the slump, bleeding, setting time, and compressive strength of concrete. 

## 2. Experimental Program

The applicability of the LCDBA was investigated with the aim of developing practical applications as a sustainable and alternative alkali activator for GGBFS blended cement concrete. The activation effects of the LCDBA were characterized using GGBFS blended cement paste. In addition, the fresh and hardened properties of GGBFS blended cement concrete incorporating LCDBA were investigated.

### 2.1. Raw Materials

**Figure 1:**
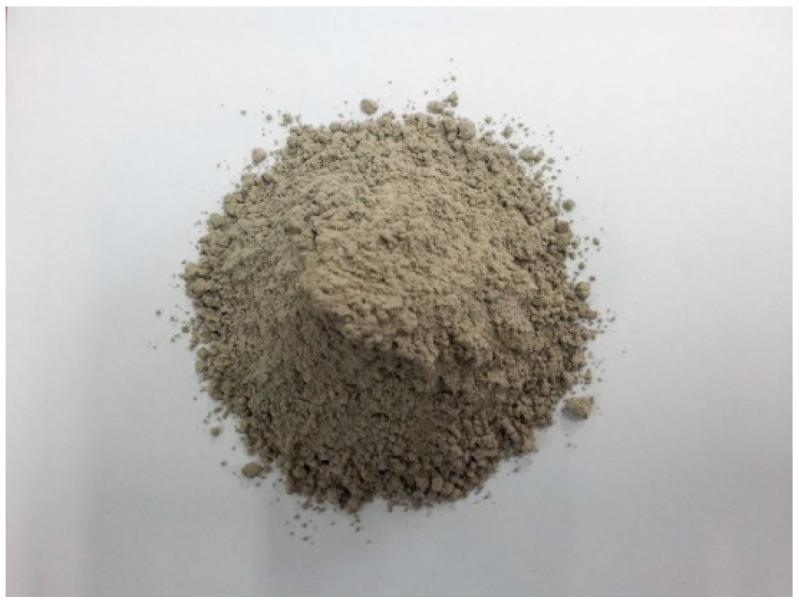
Image of LCDBA used in this study.

**Figure 2:**
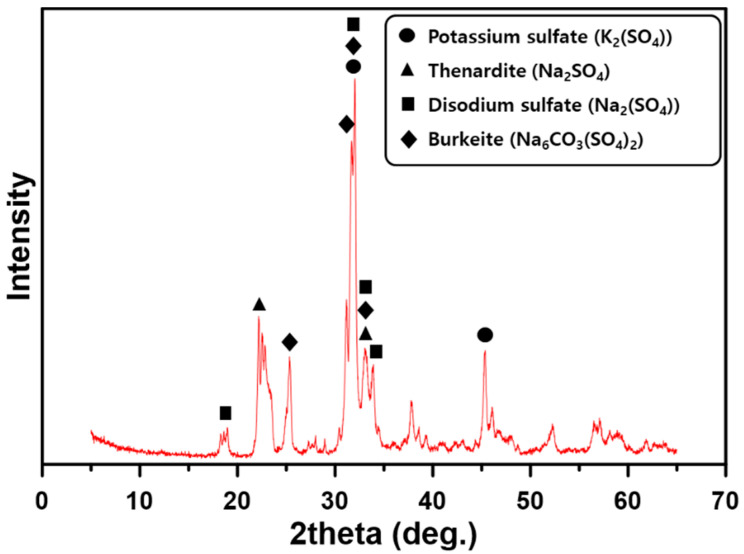
XRD patterns of LCDBA.

**Figure 3:**
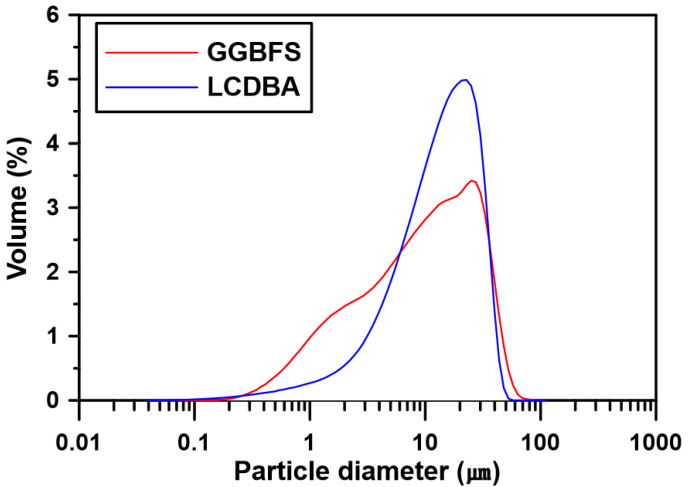
Particle size distribution of GGBFS and LCDBA.

OPC (ASTM C 150 Type I) and GGBFS were used as binders to prepare the paste and concrete and their chemical compositions are summarized in Table 1. The OPC and GGBFS that were used had a Blaine specific surface of 339 m^2^/kg and 449 m^2^/kg and a density of 3150 kg/m^3^ and 2910 kg/m^3^, respectively. The major chemical components of GGBFS were CaO, SiO_2_, and Al_2_O_3_, as shown in Table 1. The basicity coefficient (Kb = (CaO + MgO)/(SiO_2_ + Al_2_O_3_)) was 0.977, which is similar to the neutral value of 1.0 for ideal alkali activation [19]. The hydration modulus, according to a formula proposed in the literature (HM = (CaO + MgO + Al_2_O_3_)/SiO_2_) of GGBFS, was 1.92. This was higher than the value of 1.4, which is required for good hydration properties of GGBFS [19]. The LCD by-product of the refining process (see Figure 1), LCDBA—the chemical composition of which is given in Table 1—had a Blaine specific surface of 770 m^2^/kg and a density of 2540 kg/m^3^. XRD analysis was performed to determine the nature of LCDBA, as shown in Figure 2, which indicated that this was crystallized mainly with K_2_SO_4_ and Na_2_SO_4_. The particle size distributions of GGBFS and LCDBA are shown in Figure 3, which were measured using a laser particle size analyzer (PSA, Beckman Coulter LS 13 320, Brea, CA, USA). The overall particle size distribution of LCDBA was similar to that of GGBFS, showing a narrow distribution with the peak around 10 μm. Although LCDBA had about 1.7 times higher specific surface in comparison to GGBFS, the particle size of LCDBA was slightly larger than that of GGBFS, which indicated that LCDBA has porous microstructures resulting from the refining process.

Non-reactive river sand, with a specific gravity of 2.62, a fineness modulus of 2.77, and an absorption capacity of 1.1%, was used in preparation of all mortars and concrete. The crushed basalt aggregates were adopted as the coarse aggregate for the concrete mix in which the maximum size, specific gravity, absorption capacity, and fineness modulus were 25 mm, 2.63, 0.8%, and 6.4, respectively. In addition, a polycarboxylate-based superplasticizer with 17% solid content by weight was used to enhance particle dispersions within the mixture. 

### 2.2. Mix Proportions

The characterization of the reactivity of the binders was carried out based on XRD and TG-DTA tests using GGBFS blended cement paste with the OPC/GGBFS ratio of 55:45 and the water/binder ratio of 30%. LCDBA replaced the binder by 0%, 3%, and 5%. Table 2 shows the concrete mix proportions according to the usage of LCDBA. As in the paste mix design, the ratio of OPC and GGBFS was set to 55:45. In addition, the binder substitution ratio of LCDBA was set to 0%, 3%, and 5% in order to evaluate the effects of the LCDBA substitution level on the fresh and hardened properties of GGBFS blended cement concrete. The GGBFS blended cement concrete was designed to have the total volume of mortar in the 615 ± 5 L range and the slump of concrete in the 180 ± 25 mm range by controlling the amount of superplasticizer and the sand to total aggregate volume ratio (s/a). 

### 2.3. Experimental Methods

The reactivity of the paste with different LCDBA substitution levels was investigated through XRD and TG analysis. GGBFS blended cement pastes were cured at 20 ℃ and 90 ± 2% relative humidity for a predetermined curing duration (3, 7, and 28 days). Thereafter, the samples were ground and immersed in acetone to stop hydration and were suction filtered using an aspirator. The crushed samples that stopped hydration were ground further and powdered to particles smaller than 106 μm for the XRD and TG measurements. XRD was conducted using a D/MAX 2500V/PC (Rigaku, Tokyo, Japan) with a scan range of 5°–65° 2θ. TG was conducted using a NETZSCH STA 409 C/CD (NETZSCH, Selb, Bavaria, Germany) with a heating rate of 5 ℃/min in the 20–1000 ℃ range. 

The fresh properties of GGBFS blended cement concrete were measured using the slump, bleeding, and setting time. The slump of fresh concrete were measured in accordance with ASTM C 94 [20]. The bleeding test of fresh concrete was measured in accordance with ASTM C 232 [21] by drawing off the bleed water until cessation of bleeding. The initial and final setting times were measured using a penetration resistance apparatus in accordance with ASTM C 803 [22]. The measurements were conducted on sieved mortar samples from the mixed concrete. The fresh concrete was cast into cylindrical molds (100 × 200 mm) for the compression test. Following 24 h of air curing, these cylindrical samples were demolded and immersed into water at a temperature of 20 ± 1 ℃ for additional curing. Compressive strength tests were carried out in accordance with ASTM C 39 [23]. The strength was determined at 3, 7, 28, and 91 days of curing by averaging the tested values of the three replicates. 

## 3. Test Results and Discussions

### 3.1. XRD Analysis

**Figure 4:**
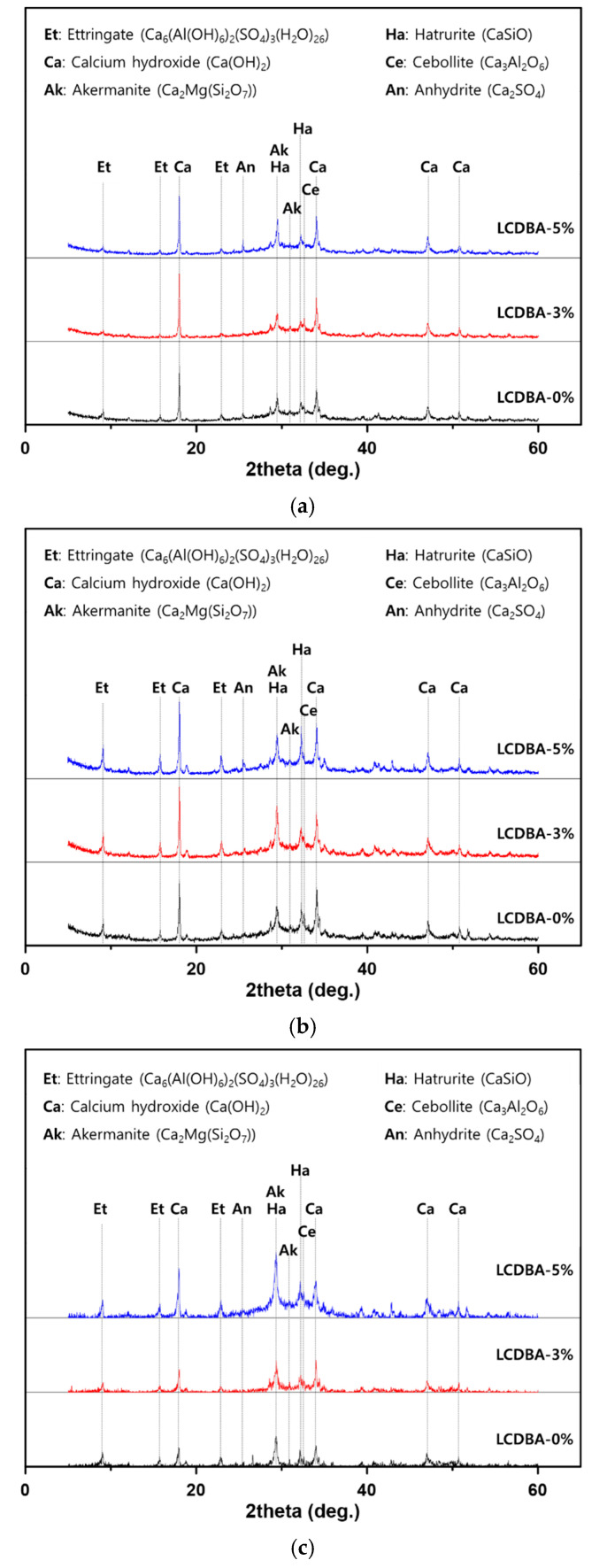
XRD results of the blended pastes at different curing ages: (**a**) 3 days, (**b**) 7 days, and (**c**) 28 days.

The diffraction patterns of the hardened pastes were analyzed by characterizing the crystal phases in order to investigate the applicability of LCDBA with high alkali content for stimulating GGBFS. The activation effect of LCDBA for GGBFS can be demonstrated by investigating the generation of hydration products for different curing ages. Figure 4 shows the results of an XRD analysis of the pastes, according to the LCDBA substitution level. 

As can be seen in Figure 3, all specimens included akermanite (Ca_2_Mg(Si_2_O_7_)) with strong peaks in the XRD analysis. It has been reported that akermanite exists in a crystalline form in raw materials and hardened GGBFS pastes [24,25] and that it can be identified more clearly for cases with LCDBA. In particular, a strong akermanite peak was observed for the 5% LCDBA series at 28 days of curing, indicating that LCDBA affects the hydration of GGBFS in the long term. 

In addition, the strong alkali compound of the cement hydration product—calcium hydroxide (Ca(OH)_2_)—is known to play a role in promoting the activation reaction of GGBFS [2]. This effect is evidenced by the XRD pattern at 3 and 28 days. For example, it can be clearly seen in the LCDBA-0% series that the higher peak of the Ca(OH)_2_ at 3 days decreases as the curing age increases through the consuming necessary for the activation of the GGBFS to form calcium-silicate-hydrate (C-S-H) gel. The pastes of the two series containing LCDBA also had high Ca(OH)_2_ at their early age and the amount decreased as the age increased. However, the decrease of Ca(OH)_2_ was smaller than that of LCDBA-0% series, although the C-S-H gel peak increased significantly. This phenomenon was the likely cause of the high alkaline LCDBA activator. In the section where 2θ is around 9°, it is known that ettringite peaks result from the hydration of cement [26]. It should be noted that the ettringite peak was less affected by the incorporation of LCDBA at 3 days, increasing with the higher LCDBA amount at 28 days. This implies that the production of ettringite is increased by the supply of SO_3_, as with the high SO_3_ content in LCDBA, at 47.1%. In addition, Mohammed and Safiullah [27] revealed that the amount of ettringite formation highly correlates with the amount of SO_3_. 

### 3.2. TG Analysis

**Figure 5:**
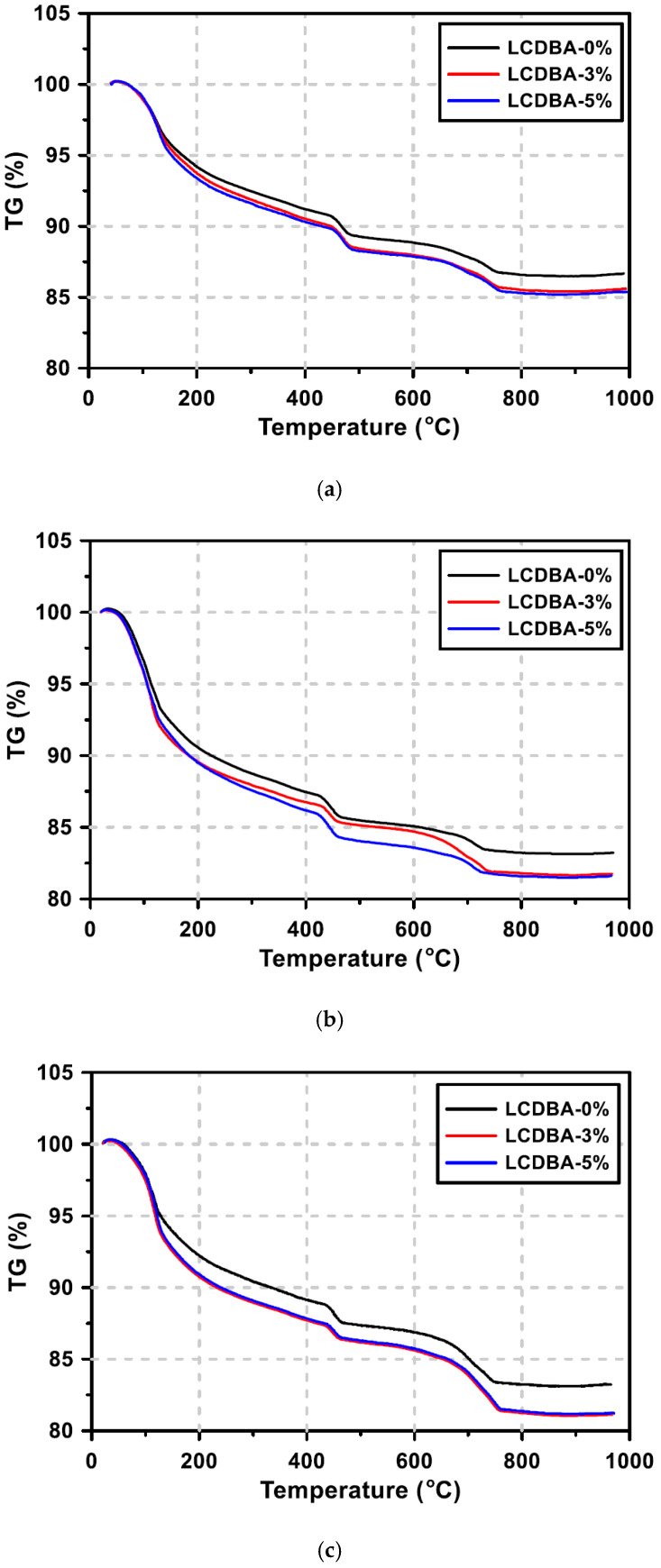
TG curves of the blended pastes at different curing ages: (**a**) 3 days, (**b**) 7 days, and (**c**) 28 days.

The degrees of hydration of GGBFS blended cement pastes were evaluated using the thermogravimetry method and the results are presented in Figure 5. The weight loss at around 100 ℃ (refer to Section I afterward) can mostly be attributed to the decomposition of ettringite and C-S-H gel [28]. The weight loss at around 450 ℃ (refer to Section II afterward) can primarily be attributed to the decomposition of Ca(OH)_2_ to CaO [29]. 

From the results for Sections I and II, it can be seen that the weight losses of the LCDBA-3% and LCDBA-5% series are more pronounced than those of the LCDBA-0% series for all curing ages. Therefore, the large mass change in Section I implies that a large amount of C-S-H gel initially forms due to the incorporation of LCDBA that contains abundant alkali compounds that promote the reactivity of GGBFS. The large mass change in Section II can be explained that the cement hydration product, Ca(OH)_2_, was relatively less consumed in the LCDBA-3% and LCDBA-5% mixtures due to the additional supply of alkaline compounds by LCDBA. In the meantime, it should be noted that the differences in mass change between the pastes with LCDBA and without LCDBA at 28 days were relatively large in Section I in comparison to those in Section II. This might be attributed to the fact that, as curing age increases, the alkali components supplied for the pozzolanic reaction of GGBFS in the LCDBA-3% and LCDBA-5% series were also consumed, resulting in a Ca(OH)_2_ decrease and reaching similar remaining levels of Ca(OH)_2_ as those of the LCDBA-0% series at 28 days of curing. 

### 3.3. Workability

**Figure 6:**
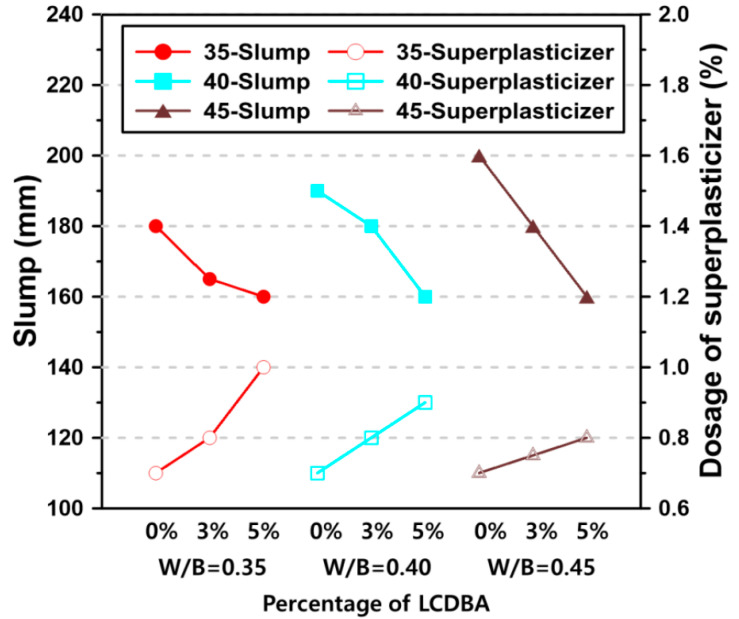
Slump of the blended concrete with different water/binder ratios and LCDBA amount (solid markers and hollow markers stand for slump values and dosage of superplasticizer, respectively).

The slump test is widely used and the most well-known test to assess the workability of concrete. In this study, the effect of the LCDBA usage on workability was evaluated using the slump test. Figure 6 shows the slump values and the superplasticizer dosage of the blended concrete, indicating that all tested series met the target slump level of 180 ± 25 mm. For all concrete series without LCDBA, the superplasticizer amount was fixed at 0.7%. The figure shows that as the water/binder ratio increases the slump increases by 10 mm per 0.05 of water/binder ratio. The blended concrete that contained 3% and 5% LCDBA tended to decrease in slump even though the dosage of superplasticizer increased. For mixtures with 5% LCDBA, all slump results were the same, 160 mm, and the corresponding superplasticizer amounts were 1.0%, 0.9%, and 0.8%, for the water/binder ratios of 0.35, 0.40, and 0.45, respectively. Therefore, the increase in the required superplasticizer amount for maintaining a similar level of workability implies that the porous LCDBA absorbs the mix water during the mixing and thus degrades the workability of the blended concrete.

### 3.4. Bleeding

**Figure 7:**
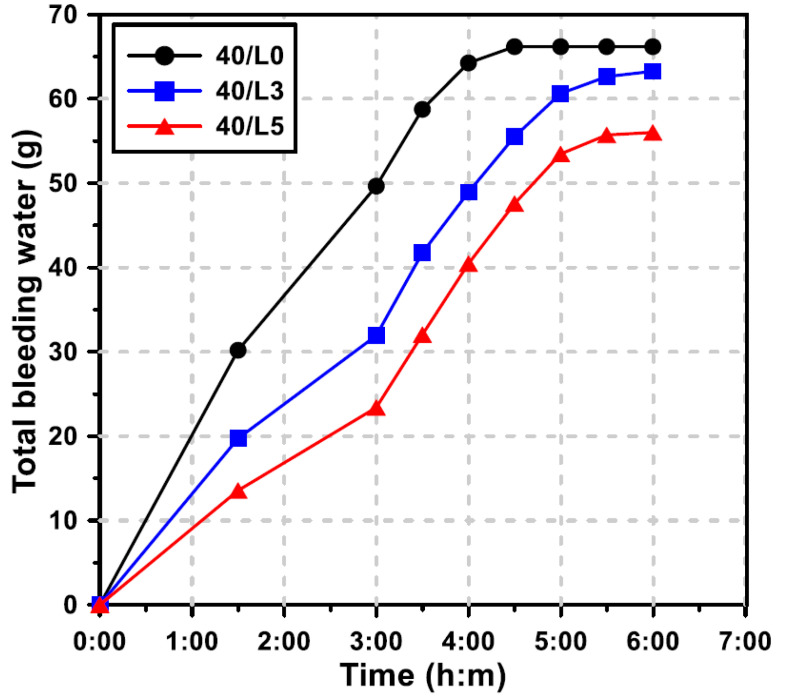
Effect of the LCDBA replacement level on bleeding (W/B = 0.40).

The bleeding of concrete, a necessary part of the life of concrete, was considered to occur when the mix water would raise to the surface of freshly placed concrete. It has been reported that the bleed rate and capacity of GGBFS blended cement concrete highly depends on the GGBFS replacement level and the water/binder ratio [30]. Figure 7 shows the development of bleeding over elapsed time of concrete for the water/binder ratio of 0.40, as a function of the LCDBA replacement level. The ending point and the slope of the curves indicate the bleeding capacity of the mixtures and the bleeding rate, respectively. It should be pointed out from the figure that the bleeding capacity decreases as the LCDBA replacement level increases. In particular, the mixture without LCDBA (the black curve) showed the highest bleeding rate for the first four hours and then it quickly reached the highest value. On the other hand, the mixtures with LCDBA (the blue and red curves) showed that as the LCDBA replacement level increases the bleeding rate becomes slower for the first three hours. After the first three hours, all mixtures showed similar bleeding rates until reach the bleeding capacity. The delayed endings of the bleeding were observed for both mixtures with LCDBA in comparison to the one without LCDBA. This can be attributed to the fact that the porous LCDBA absorbed excess water during mixing because of its high specific surface area, which reduced the initial bleeding rate. In addition, the bleeding time increased as the water absorbed by the LCDBA was slowly released to the fresh mixture.

### 3.5. Setting Time

**Figure 8:**
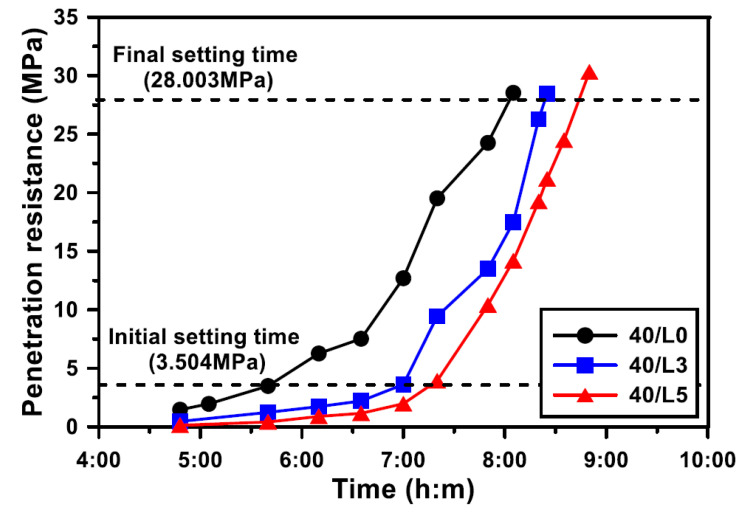
Effect of the LCDBA replacement level on penetration resistance (W/B = 0.40).

The effect of the LCDBA replacement level on the initial and final setting times of GGBFS blended cement concrete is shown in Figure 8. The setting times were defined when the penetration resistance reached the pre-defined criteria in accordance with ASTM C 803 [22]. The initial setting time of the mixture without LCDBA was 340 min but the mixtures incorporating LCDBA were delayed for more than 80 min. The delay in the initial setting time can be explained by the delay in the bleeding. Similar to the results of the initial setting time, the mixture without LCDBA showed the fastest final setting time, at 483 min, and 20- and 40-min delays were observed for the mixtures with 3% and 5% LCDBA replacement, respectively. It should be pointed out, however, that the differences of final setting time between the mixtures with and without LCDBA were smaller than those of the initial setting time. This can be attributed to the activation of GGBFS promoted by the alkali supply of LCDBA after initial setting.

### 3.6. Compressive Strength

**Figure 9:**
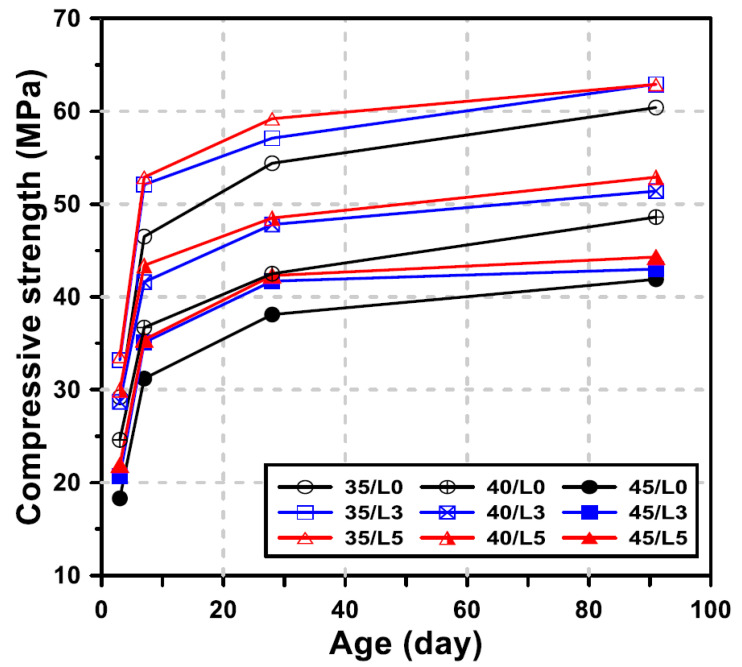
Effect of the LCDBA replacement level on compressive strength.

The compressive strength development of GGBFS blended cement concrete with different LCDBA replacement levels and with water/binder ratios at 3, 7, 28, and 91 days is shown in Figure 9. The concrete mixtures that incorporate LCDBA showed a generally higher strength than the mixture without LCDBA. In particular, the incorporation of LCDBA was more effective for early strength development and for mixtures with lower water/binder ratios.

The rates of increase in the compressive strength of the concrete incorporating LCDBA relative to the strength of concrete without LCDBA were evaluated for each curing age, with the results summarized in Table 3. At 3 days of curing, the strength improvement rates of the concrete with 3% and 5% replacement were 13.1–16.7% and 15.1–22.0%, respectively. On the other hand, the strength improvement rates of the concrete with 3% and 5% replacement at 91 days of curing were 1.4–5.8% and 4.1–8.8%, respectively. Therefore, as the replacement level of LCDBA increased, the strength increase rate became higher, while the strength increase rate gradually decreased as the curing age increased. This tendency is similar to the typical pattern of rapid strength development of GGBFS promoted by alkaline activators (see also [31,32,33]). It should be pointed out, therefore, that LCDBA could be a sustainable and alternative activator for GGBFS blended cement concrete, with similar effects as those of conventional alkaline activators, which is effective for the early-age strength development of concrete.

In order to quantitatively analyze the strength developing characteristics of GGBFS blended cement concrete incorporating LCDBA, the compressive strength prediction model suggested in ACI 209 was used. The ACI Committee 209 [34] recommends the following equation for predicting the compressive strength of concrete with time

(1) $${\left(f_{c}^{\prime }\right)}_{t}=\frac{t}{a+b\times t}{\left(f_{c}^{\prime }\right)}_{28},$$

where *a* and *b* are material constants considering the type of binders and curing methods, calculated in this research based on regression analysis using measured values. In addition, ${\left(f_{c}^{\prime }\right)}_{t}$ and ${\left(f_{c}^{\prime }\right)}_{28}$ are compressive strength at the age of *t* and 28 days, respectively. 

**Figure 10:**
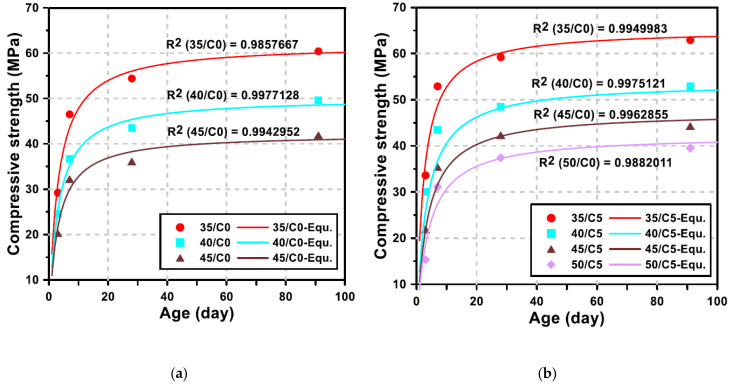
Comparison between the measured compressive strength and the regression curves for 0% or 5% LCDBA replacement series; (**a**) LCDBA-0%, (**b**) LCDBA-5%.

The concrete mixtures with 5% LCDBA replacement and without LCDBA were considered to quantitatively illustrate the effect of LCDBA on the development of the compressive strength of concrete. Figure 10 shows the compressive strength results, according to various curing ages and water/binder ratios for the concrete mixtures with 0% or 5% of LCDBA and with the regression curves based on the ACI 209 model. 

**Figure 11:**
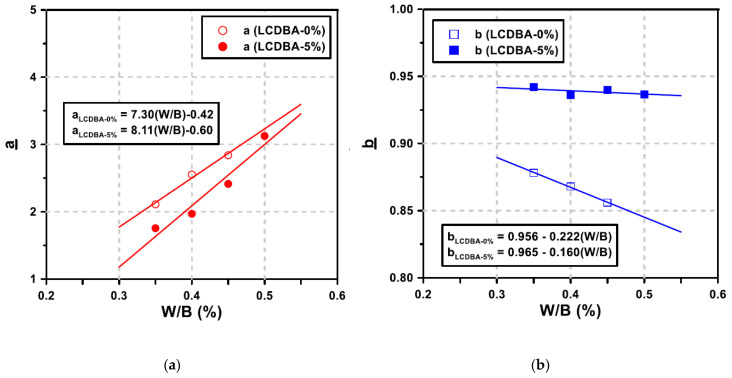
Relationship between the material constants and the W/B for 0% or 5% LCDBA replacement series, corresponding to Figure 9; (**a**) constant *a* (**b**) constant *b*.

As a result of the regression analysis of the compressive strength, according to the ACI 209 model, the R-squared values for the mixtures with and without LCDBA were higher than 0.985, indicating a high goodness of fit. With the high goodness of fit, the material constants *a* and *b* were used to further analyze the strength development patterns of the tested concrete. The calculated material constants *a* and *b* are illustrated in Figure 11, where the constants *a* and *b* considerably correlate with the strength development of concrete at early age and at long-term age, respectively [35]. Mathematically, *a* is inversely proportional to the initial strength development and *b* is inversely proportional to the increase in compressive strength according to the curing age. As shown in the figure, *a* and *b* tend to decrease and increase, respectively, as the water/binder ratio increases. As the water/binder ratio decreases, the initial strength development rate becomes high, which leads to a decrease in *a*. On the other hand, the material constant *b* is related to the long-term strength development, where the lower *b* value stands for the higher long-term strength increment. The constant *b* values for the mixtures with LCDBA were similar, regardless of the water/binder ratio, and higher than those for the mixtures without LCDBA. The higher *b* values for the mixture with LCDBA were attributed to the higher initial strength development activated by LCDBA and to relatively less long-term strength development. This is similar to the strength development characteristics of GGBFS and/or fly ash blended cement concrete activated using conventional alkali activators. Therefore, it can be concluded that LCDBA is an effective and sustainable alternative to alkali activators for GGBFS blended cement concrete.

## 4. Conclusions

This experimental study evaluates the LCD by-product of the refining process, LCDBA, as an alternative and sustainable alkaline activator for GGBFS blended cement concrete. To investigate the applicability of this the alternative activator, the tested experimental parameters were set at three LCDBA replacement levels and four water/binder ratios. The activation effects were characterized based on XRD and TG analyses using GGBFS blended cement paste. The fresh and hardened properties of GGBFS blended cement concrete incorporating LCDBA were investigated using slump, bleeding, setting time, and compressive strength tests. The key observations and findings of this research can be summarized as follows:

(1) The effectiveness of LCDBA as an alternative and sustainable alkaline activator for GGBFS blended cement concrete was demonstrated for the first time in this study. The formations of akermanite and ettringite, as well as the consumption of Ca(OH)_2_ due to the stimulation of GGBFS hydrations with LCDBA as days of curing increase, were characterized using XRD analysis. This finding highlights the role of LCDBA as an activator for GGBFS blended cement concrete by providing the essential chemical evidence.
(2) The thermogravimetric analysis was employed to evaluate the degrees of hydration. The results highlight that a large amount of C-S-H gel was initially formed and that the cement hydration product, Ca(OH)_2_, was relatively less consumed due to the incorporation of LCDBA that contains abundant alkali compounds that promote the reactivity of GGBFS. As curing age increased, the remaining amount of Ca(OH)_2_ in the pastes with LCDBA became similar as to the one in the pastes without LCDBA, which is another implication of the relatively less long-term strength development.
(3) It is identified from this research that by incorporating LCDBA, the fresh GGBFS blended cement concrete showed degraded workability, delayed bleeding ends, reduced bleeding capacity, and delayed setting times, attributed to the porous nature of LCDBA that led to mix water absorption.
(4) The series of compressive strength tests conducted in this research concluded that LCDBA was an effective alkaline activator for GGBFS blended cement concrete, showing early-age strength developing characteristics, especially for mixtures with lower water/binder ratios. The compressive strength model, suggested by the ACI Committee 209, also highlighted the early-age strength developing characteristics of the blended concrete activated with LCDBA.

The results obtained in this study provide a simple, yet effective and practical, means of reusing an industrial by-product as an alternative alkaline activator for GGBFS blended concrete. Further studies are, however, necessary to determine its long-term durability and dimensional stabilities, such as shrinkage and creep.

## Figures and Tables

**Table 1:** Chemical composition of OPC, GGBFS, and LCDBA (wt.%).

Element	SiO_2_	Al_2_O_3_	Fe_2_O_3_	CaO	MgO	SO_3_	K_2_O	Na_2_O	LOI
OPC	19.5	5.6	3.5	61.5	3.8	2.5	1.1	0.1	2.5
GGBFS	32.6	15.5	0.5	42.2	4.6	3.3	0.5	0.2	-
LCDBA	4.3	-	0.2	10.0	0.5	47.4	4.3	30.4	3.0

**Table 2:** Mix proportions of concrete.

Mixture	W/B (%)	s/a (%)	Unit Weight (kg/m^3^)						Super-Plasticizer (%)
			Water	Binder			Fine Aggregate	Coarse Aggregate	
				OPC	GGBFS	LCDBA			
35/L0	35	47.5	160	251	206	-	814	903	0.70
35/L3	35	46.0	160	244	200	14	788	929	0.80
35/L5	35	45.0	160	239	195	23	770	945	1.00
40/L0	40	49.0	160	220	180	-	864	903	0.70
40/L3	40	48.0	160	213	175	12	846	920	0.80
40/L5	40	47.5	160	209	171	20	837	928	0.97
45/L0	45	50.0	160	196	160	-	901	904	0.70
45/L3	45	49.0	160	190	155	11	882	922	0.75
45/L5	45	48.0	160	186	152	18	864	940	0.80
50/L5	50	49.0	160	167	137	16	897	937	0.70

**Table 3:** Rate of increase in the compressive strength of concrete incorporating LCDBA.

Mixture	Curing Age (%)			
	3 Days	7 Days	28 Days	91 Days
35/L3	13.7	12.0	5.0	4.1
35/L5	15.1	13.8	8.8	4.1
40/L3	16.7	13.4	12.5	5.8
40/L5	22.0	18.3	14.1	8.8
45/L3	13.1	9.0	1.5	1.4
45/L5	19.7	9.9	2.9	4.5
